# Climate-Driven Changes in the Nutritional Value and Food Safety of Legume Seeds

**DOI:** 10.3390/nu17233703

**Published:** 2025-11-26

**Authors:** Mateusz Labudda, Wesley Borges Wurlitzer, Tomasz Niedziński, Julia Renata Schneider, Jakub Frankowski, Szymon Florczak, Ewa Muszyńska, Mirosława Górecka, Monika Tomczykowa, Beata Prabucka, Anna Rybarczyk-Płońska, Wojciech Makowski, Maria Goreti de Almeida Oliveira, Katarzyna Leszczyńska, Iwona Morkunas, Noeli Juarez Ferla, Michał Tomczyk

**Affiliations:** 1 Department of Biochemistry and Microbiology, Institute of Biology, Warsaw University of Life Sciences—SGGW, ul. Nowoursynowska 159, 02-776 Warsaw, Polandbeata_prabucka@sggw.edu.pl (B.P.); anna_rybarczyk-plonska@sggw.edu.pl (A.R.-P.); 2 Laboratory of Acarology, Tecnovates, University of Vale do Taquari—Univates, Lajeado 95914-014, RS, Brazil; wesleeywurlitzer@gmail.com (W.B.W.); julia.schneider4@universo.univates.br (J.R.S.); njferla@univates.br (N.J.F.); 3 Postgraduate Program in Biotechnology, University of Vale do Taquari—Univates, Lajeado 95914-014, RS, Brazil; 4 Bioagro—Institute of Biotechnology Applied to Agriculture/INCT-Plant-Pest Interactions, Federal University of Viçosa—UFV, Viçosa 36570-900, MG, Brazil; malmeida@ufv.br; 5 Division of Agricultural and Environmental Chemistry, Institute of Agriculture, Warsaw University of Life Sciences—SGGW, ul. Nowoursynowska 159, 02-776 Warsaw, Poland; tomasz_niedzinski@sggw.edu.pl; 6 School of Medical and Health Sciences, VIZJA University, ul. Okopowa 59, 01-043 Warsaw, Poland; j.frankowski@vizja.pl; 7 Department of Botany and Plant Physiology, Institute of Biology, Warsaw University of Life Sciences—SGGW, ul. Nowoursynowska 159, 02-776 Warsaw, Poland; ewa_muszynska@sggw.edu.pl (E.M.); miroslawa_gorecka1@sggw.edu.pl (M.G.); 8 Department of Organic Chemistry, Faculty of Medicine with the Division of Dentistry and Division of Medical Education in English, Medical University of Białystok, ul. Mickiewicza 2a, 15-222 Białystok, Poland; monika.tomczyk@umb.edu.pl; 9 Department of Botany, Physiology and Plant Protection, Faculty of Biotechnology and Horticulture, University of Agriculture in Krakow, al. Mickiewicza 21, 31-120 Kraków, Poland; wojciech.makowski@urk.edu.pl; 10 Department of Medical Microbiology and Nanobiomedical Enginnering, Faculty of Medicine with the Division of Dentistry and Division of Medical Education in English, Medical University of Białystok, ul. Mickiewicza 2c, 15-222 Białystok, Poland; 11 Department of Plant Physiology, Faculty of Agriculture, Horticulture and Biotechnology, Poznań University of Life Sciences, ul. Wołyńska 35, 60-637 Poznań, Poland; iwona.morkunas@up.poznan.pl; 12 Postgraduate Program in Environment and Development, University of Vale do Taquari—Univates, Lajeado 95914-014, RS, Brazil; 13 Department of Biology and Pharmacognosy, Faculty of Pharmacy with the Division of Laboratory Medicine, Medical University of Białystok, ul. Mickiewicza 2a, 15-222 Białystok, Poland; michal.tomczyk@umb.edu.pl

**Keywords:** antioxidants, bioactive compounds, climate change, Fabaceae, food safety, integrated pest management, legumes, nutrition, pests, public health

## Abstract

**Background/Objectives:** Leguminous plants (Fabaceae) are essential for global food and nutritional security due to their high protein content, bioactive compounds, and ecological role in nitrogen fixation. However, climate change poses significant threats to their productivity, quality, and safety. This review aims to summarize the nutritional, biochemical, and health-related importance of legumes, while highlighting the effects of climate change—particularly heat stress and pest pressure—on their nutritional value and public health implications. **Methods:** This review is based on an integrative literature review drawing on scientific databases including Web of Science, Scopus, ScienceDirect, Google Scholar, and PubMed (March–October 2025). The relevant literature on climate change, legume composition, stress physiology, pest–plant interactions, and nutrition- and health-related outcomes was identified using targeted search terms. Evidence from diverse study types was synthesized to provide a broad, interdisciplinary perspective rather than a systematic assessment. **Results:** Legume seeds are rich in proteins, complex carbohydrates, fibers, and essential fatty acids, and contain valuable phytochemicals, including polyphenols, carotenoids, saponins, and bioactive peptides, with antioxidant, anti-inflammatory, and cardioprotective effects. Nevertheless, elevated CO_2_ levels and temperature stress can reduce protein, iron, and zinc contents, while altering phenolic and isoflavone profiles. Simultaneously, warming enhances pest proliferation and fungal contamination, increasing mycotoxin exposure and associated health risks. Integrated pest management (IPM) strategies, particularly those emphasizing biological control, show promise in mitigating these risks while ensuring sustainable legume production. **Conclusions:** Safeguarding the nutritional and ecological value of legumes under changing climatic conditions requires coordinated efforts across plant breeding, agronomy, and food science. Enhancing thermotolerance and pest resistance, reducing pesticide use through IPM, and valorizing legume by-products are key to preserving food safety and human health. Legumes, thus, represent both a challenge and an opportunity in achieving resilient, climate-smart nutrition systems for future generations.

## 1. Introduction

**Figure 1:**
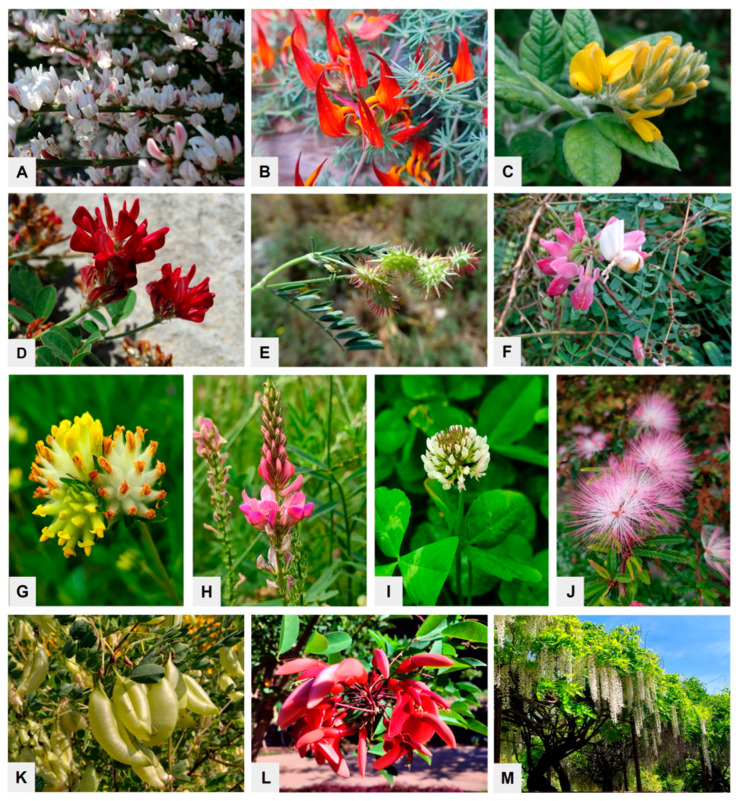
The diversity of Fabaceae representatives: (**A**–**C**) endemic plants like *Spartocytisus supranubius* (**A**), *Lotus berthelotii* (**B**), *Teline nervosa* (**C**); (**D**–**I**), economically important species like *Hedysarum coronarium* (**D**), *Onobrychis caput-galli* (**E**), *Coronilla viminalis* (**F**), *Anthyllis vulneraria* (**G**), *Onobrychis vicifolia* (**H**), *Trifolium repens* (**I**); (**J**–**M**), ornamental woody plants like *Albizia julibrissin* (**J**), *Colutea arborescens* (**K**), *Erythrina crista-galli* (**L**), and *Wisteria floribunda* (**M**).

The Fabaceae (also known as Leguminosae) plant family ranks as the third largest among angiosperms, encompassing over 19,000 species found across a diverse array of habitats worldwide. Many of its wild representatives are cosmopolitan (e.g., *Trifolium repens*); others are globally cultivated for various utility purposes (e.g., *Albizia julibrissin*, an ornamental plant in warm and temperate zones); and still others belong to endemic plants with a strictly defined geographic area (e.g., *Spartocytisus supranubius*, *Lotus berthelotii*, *Teline nervosa*, growing in the Canary Islands of Spain) (Figure 1A–C). Members of this family exhibit considerable morphological diversity, ranging from herbaceous annuals to woody lianas and massive trees [1]. A hallmark of legumes is their capacity to form symbiotic relationships with nitrogen-fixing bacteria (rhizobia) that reside in root nodules. This unique symbiosis enables the biological conversion of atmospheric nitrogen into forms that are accessible to plants, thereby significantly improving soil fertility. Thus, legumes are vital contributors to both natural ecosystems and agricultural production systems [2,3].

Legumes play a pivotal role in global agriculture, encompassing key cultivated crops for human nutrition such as soybean (*Glycine max*), common bean (*Phaseolus vulgaris*), chickpea (*Cicer arietinum*), faba bean (*Vicia faba*), pea (*Pisum sativum*), and lentil *(Lens culinaris*). The grain legumes are cultivated on approximately 81 million hectares, and their global production exceeds 92 million tons [4]. These species are essential sources of plant-based proteins, dietary fiber, vitamins, and minerals. In addition to their nutritional benefits for humans, legumes also supply high-protein fodder for livestock and enhance sustainable farming practices through crop rotation and green manuring. Their ability to fix nitrogen reduces dependency on mineral fertilizers, aligning with agroecological principles and climate-smart agricultural strategies, thus supporting the production of a better-quality crop, above all, with a lower toxicological burden from heavy metals [5,6].

Among the species of economic importance, *T. repens*, *Trifolium hybridum*, *Lotus corniculatus*, *Lupinus angustifolius*, and *Onobrychis viciifolia* or *O. caput-galli* should be mentioned (Figure 1D–I). Such plants can be used not only as melliferous, fodder, or fertilizer plants, but also to increase the content of organic matter and improve the soil structure [7]. Additionally, some Fabaceae representatives, such as *Coronilla viminalis*, *Anthyllis vulneraria*, or *Medicago sativa*, show high resistance to harsh habitat conditions, including soil poverty, drought, or metal and organic contamination, which makes them essential pioneers with high ecological value to the reclamation of degraded areas [8,9,10].

The Fabaceae family also comprises numerous ornamental species. Some of them, such as *Albizia*, *Wisteria*, *Laburnum*, *Colutea*, and *Erythrina*, are examples of woody plants that can be cultivated in households and urban areas (Figure 1J–M). Through the beauty of their foliage, flowers, fruits, and even habits, they contribute to improving the quality of the living environment and people’s health. Beyond aesthetic value, established green spaces serve as natural regulators of temperature, act as wind and sound buffer barriers, filter the air, and prevent soil erosion [11]. Recent research highlights that the lack of stable plant communities in neighborhoods leads to elevated stress, mental health disorders, and antisocial behavior in the urban population [12,13]. Many members of the Fabaceae family can be helpful in this matter, and their incorporation into urban spaces and landscapes promotes both ecological balance and public well-being.

Selected species representing various functional groups of the Fabaceae family are presented in Figure 1. Undoubtedly, due to such enormous morphological, ecological, and functional diversity, the integration of legumes into diverse aspects of human activity is both feasible and potentially transformative, offering significant support to sustainable agriculture, ecological integrity, and the promotion of human health. Accordingly, this article presents only a part of the legume’s multifaceted contributions, examining its nutritional value in the context of ongoing climate change.

### Literature Search Methodology

An integrative review was conducted to gather relevant literature through a structured search methodology, complemented by critical analysis. The literature search, conducted from March to October 2025, encompassed various scientific databases, including Web of Science, Scopus, ScienceDirect, Google Scholar, and PubMed. Key terms used in this search included “climate change”, “global warming”, “legumes”, “Fabaceae”, “bioactive compounds”, “nutritional properties”, “health benefits”, “pest management”, “agriculture”, “agronomy”, “horticulture”, “pharmacognosy”, “medicinal plants”, “plant defense”, “heat stress” and “integrated pest management” in addition to other related term. To maintain high scientific standards and relevance, the inclusion criteria were intentionally limited to research and review articles published in the English language. As a result, materials such as popular science articles, theses, and editorials were excluded from consideration. Following an initial screening, a detailed content analysis was conducted to identify articles that provide empirical data, theoretical insights, or noteworthy advancements in agronomy, human health, and nutrition. Although the methodology employed does not strictly adhere to the framework of a systematic review, it was intentionally designed to provide a comprehensive overview of the existing literature. The focus was on exploring the potential role of legumes in targeted nutrition and phytomedicine, addressing global nutritional needs in consideration of a growing population, with integrated pest management as a key component. Consequently, the choice of this topic and the time limit were made with careful consideration to create a literature review that stands as a valuable reference for future research initiatives. This review provides an integrated overview of how climate change affects the nutritional quality, biochemical properties, pest vulnerability, and food safety of legume seeds, drawing strength from its interdisciplinary scope and extensive literature base; however, as an integrative review, it may be subject to selection bias, and the rapidly evolving nature of climate–crop–pest interactions, the focus on major legume species, and limited quantitative data on nutrient losses and food safety risks constrain the generalizability and precision of some conclusions.

## 2. Nutritional and Bioactive Composition of Legume Seeds and Their By-Products

Legumes are widely acknowledged for their substantial nutritional value and health-promoting attributes. In addition to their function as staple foods, the products of legume processing, particularly hulls and seed coats, are increasingly recognized for their rich content of bioactive compounds. These compounds hold significant potential for applications in functional foods and nutraceuticals, thereby enhancing the overall value of legumes in the health and wellness industry [14]. In addition to their nutritional and bioactive properties, legume seeds contain antinutritional factors that are also considered bioactive constituents, including lectins, phytic acid, alkaloids (such as quinolizidine and pyrrolizidine alkaloids), amines, and cyanogens. Certain antinutritional factors, which may exhibit undesirable, indigestible, or toxicological properties, can be reduced or eliminated through plant genotype selection or postharvest and thermal processing methods, including dehulling, soaking, germination, extraction, boiling, leaching, and fermentation. These substances are typically present in small quantities and do not constitute a food safety hazard [15].

### 2.1. Macronutrients and Micronutrients

Legume seeds are excellent sources of plant-based protein (15–32%), complex carbohydrates (33–46%), and dietary fiber (up to 12%), while being low in fat, except for oil-rich species like soybeans and peanuts [16,17]. They also provide essential micronutrients, such as iron (2.2–9 mg/100 g), zinc (1.7–6.4 mg/100 g), and calcium (46–162 mg/100 g), with variations depending on the species and cultivar [14].

### 2.2. Polyphenolic Compounds

Polyphenols are among the most abundant and studied bioactive compounds in legume seeds, including flavonoids (e.g., kaempferol, quercetin), isoflavones (e.g., genistein, daidzein), phenolic acids (e.g., gallic, ferulic, *p*-coumaric), and condensed tannins/proanthocyanidins (catechins), which are often concentrated in the seed coat and hulls [15,16,18]. Anthocyanin compounds have also been identified. These polyphenolic compounds exhibit antioxidant, anti-inflammatory, and anticancer properties. For example, lentil and pea hulls contain both soluble and insoluble-bound phenolics, which contribute to their radical-scavenging capacity and modulation of oxidative stress pathways [14].

### 2.3. Carotenoids and Tocopherols

Legumes also contain carotenoids such as lutein and β-carotene, which are important for eye health and antioxidant defense [14,19]. Tocopherols (vitamin E homologues), particularly γ- and δ-tocopherol, are found in high concentrations in soybeans and lentils, contributing to cellular protection against lipid peroxidation [14,19].

### 2.4. Phytosterols and Saponins

Other lipophilic compounds include phytosterols and saponins, which are found in the seeds of legume species. The first group of biologically active substances is represented, in particular, by β-sitosterol, campesterol, and stigmasterol, which are structurally similar to cholesterol and are found in various plant species. The second group of compounds, saponins, occurs mainly in free aglycone forms (sapogenins). Saponins, which have been studied as the primary compounds in legume seeds, include soyasaponin I and dehydrosoyasaponin I [20].

### 2.5. Essential Fatty Acids

Although legume seeds are generally low in fat, they provide essential fatty acids, including linoleic (omega-6) and α-linolenic (omega-3) acids. The omega-6/omega-3 ratio varies among species, with kidney and black beans showing favorable ratios (<1), while chickpeas and faba beans exhibit higher ratios (>14). These fatty acids are vital for cardiovascular and neurological health [14,21].

### 2.6. Carbohydrates Including Dietary Fiber, Resistant Starch, Oligosaccharides and Gut Health

Generally, carbohydrates and many derivatives of this group of compounds are important constituents of legume seeds. Many of these species are rich in dietary fibers, starch, or oligosaccharides (raffinose, stachyose, ciceritol, verbascose). Dietary fiber, particularly derived from legume hulls, plays a crucial role in maintaining gut health. It promotes intestinal motility, supports the growth of beneficial microbiota, and serves as a carrier for polyphenols. Upon bacterial fermentation in the colon, fiber-bound phenolics are released and metabolized into bioactive compounds that contribute to systemic health. Pea and lentil hulls, for instance, have been shown to enhance intestinal barrier function and reduce inflammation in both in vitro and in vivo models [14,22,23].

### 2.7. Bioactive Peptides and Protein Hydrolysates

Legume-derived proteins and peptides exhibit a range of bioactivities, including antihypertensive, antidiabetic, and lipid-lowering effects. Protein hydrolysates from beans, chickpeas, and soy have demonstrated inhibitory activity against enzymes such as α-amylase, α-glucosidase, and dipeptidyl peptidase IV, which are relevant to the management of type 2 diabetes and obesity [14,24].

### 2.8. Valorization of By-Products

Processing by-products such as seed coats and hulls, which account for 8–20% of seed mass, is often discarded or used as low-value animal feed. However, these fractions are rich in polyphenols, fiber, and residual protein, making them ideal candidates for upcycling into functional food ingredients or nutraceuticals. Their incorporation into food systems aligns with circular economic principles, offering a sustainable strategy to reduce agricultural and food waste while enhancing dietary quality [14,25].

## 3. Ethnobotanical and Ethnopharmacological Perspectives on Legumes in Health

Ethnobotanical studies conducted in Africa, Asia, Europe, and the Americas repeatedly highlight Fabaceae species as essential sources of remedies for various health concerns, including gastrointestinal, respiratory, circulatory, and inflammatory disorders. This prominence can be attributed to their widespread distribution and the diverse array of secondary metabolites they produce. These metabolites, which include saponins, flavonoids, isoflavones, polysaccharides, alkaloids, and anthraquinones, are responsible for numerous biological activities that benefit human health [26].

*Glycyrrhiza glabra*, commonly referred to as licorice, is one of the most thoroughly studied medicinal legumes. Its roots are rich in triterpenoid saponins, particularly glycyrrhizin, as well as a variety of flavonoids [27]. These compounds are responsible for the herb’s anti-inflammatory, hepatoprotective, antiviral, and expectorant properties, which have been documented in Ayurvedic, Unani, and Traditional Chinese Medicine. Clinical studies have confirmed the gastroprotective and immunomodulatory benefits of these compounds; however, they also highlight potential mineralocorticoid-like side effects associated with prolonged use [28]. Another significant herb in Asian traditional medicine is *Astragalus membranaceus*. Its roots contain polysaccharides, astragalosides, and flavonoids that exhibit immunomodulatory, antioxidant, and cardioprotective effects, making it useful for addressing respiratory and urinary disorders [29]. In contrast, *Senna alexandrina* contains anthraquinone glycosides, which are responsible for its well-known stimulant laxative action. This property is recognized in modern pharmacopeias but comes with strict dosage guidelines to mitigate the risk of long-term adverse effects [30]. Other genera, such as *Trifolium*, *Medicago*, and *Saraca*, are prominently featured in regional ethnobotanical records for their use in treating menopausal symptoms, inflammatory conditions, and various infectious diseases [26,31].

The chemical diversity found in the Fabaceae not only accounts for their extensive therapeutic applications but also influences ecological interactions with insects, which, in the era of a dynamically changing climate, may pose a serious problem for the quality and yield of legumes. Compounds such as saponins and triterpenoids act as deterrents or toxins to various herbivorous insects. For example, strips of licorice planted along the edges of cotton fields support generalist arthropod predators, enhancing the biological control of aphids throughout the entire cotton-growing season [32]. Additionally, these compounds provide anti-inflammatory benefits to humans [28]. Isoflavones and related flavonoids, prevalent in *Trifolium* and other legumes, act as phytoestrogens in humans and simultaneously affect plant defense signaling and the feeding behavior of insects. Additionally, polysaccharides derived from *A*. *membranaceus* contribute to immunomodulatory effects and can alter microbial communities [29]. Anthraquinones found in *S*. *alexandrina* not only promote laxation in humans but also impart bitterness, serving as a deterrent to herbivores [30].

Ethnopharmacological analyses indicate that the leaves and roots of plants are the most frequently utilized parts, with oral administration being the prevailing method of delivery [26,31]. Local healers often select abundant native legumes for various therapeutic applications, making these species particularly valuable for ecological and entomological research. The convergence of traditional medicinal significance and ecological abundance suggests that ethnobotanical data can inform the discovery of plant extracts or isolated metabolites with insecticidal or insect-repellent properties, a strategy that has been advocated in several recent surveys [26,31]. Despite their promise, certain medicinal Fabaceae raise safety concerns that need to be addressed in translational research. Chronic consumption of licorice can lead to pseudo-hyperaldosteronism, resulting in hypertension and hypokalemia [28], while prolonged use of anthraquinone laxatives may result in dependency or changes to the intestinal mucosa [30].

Bioprospecting ethnobotanically significant Fabaceae species offers new opportunities for adapting agriculture to climate change. Traditionally used medicinal and utility plants, rich in secondary metabolites such as saponins, isoflavones, and alkaloids, can serve as sources of genes and bioactive compounds that support tolerance to heat, drought, and pest pressure. Many of these substances play a dual role, acting as pharmacologically relevant phytochemicals while simultaneously serving as natural repellents or toxins against herbivorous insects. Integrating ethnobotanical data into breeding programs and research on biological pest control may accelerate the identification of novel climate-resilient traits and compounds with potential applications in integrated pest management strategies. This approach aligns with the principles of sustainable agriculture and biodiversity utilization to ensure food security under changing environmental conditions.

## 4. Molecular, Biochemical, and Physiological Responses of Legumes to Climate Change: Implications for Human Nutrition and Food Security

### 4.1. Heat Stress Alters Legume Metabolism

Like many other crops, leguminous plants are particularly vulnerable to elevated temperatures, especially during their reproductive development [33]. Heat stress induces extensive transcriptional reprogramming. Heat shock transcription factors (HSFs) play a crucial role in orchestrating the expression of key heat shock proteins (HSPs) and small HSPs, which help maintain protein structure and function during stressful conditions. Transcriptome analyses have highlighted the significant roles of small RNAs and epigenetic modifications in the dynamic regulation of genes responsive to heat stress [34,35,36,37].

Heat stress disrupts cellular homeostasis primarily by inducing the excessive accumulation of reactive oxygen species (ROS), including the superoxide anion (O_2_^•−^), hydrogen peroxide (H_2_O_2_), and the hydroxyl radical (^•^OH), which can cause damage to lipids, proteins, and nucleic acids [38]. Simultaneously, plants produce reactive nitrogen species (RNS), such as nitric oxide (NO), and reactive sulfur species (RSS), particularly hydrogen sulfide (H_2_S), which act as crucial secondary messengers in heat-induced signaling pathways [39,40,41]. The interplay between ROS, RNS, and RSS is vital for activating antioxidant systems and modulating gene expression during heat stress. To mitigate oxidative damage, legumes enhance both their enzymatic and non-enzymatic antioxidant defenses. Key enzymes such as superoxide dismutase (SOD), catalase (CAT), ascorbate peroxidase (APX), and glutathione reductase (GR) are typically upregulated. Additionally, the accumulation of antioxidant metabolites, including ascorbic acid, glutathione, proline, and flavonoids, contributes to restoring redox balance and minimizing cellular injury [42,43,44,45,46,47].

Hormonal regulation is crucial for the adaptation of legumes to thermal stress [48]. Abscisic acid (ABA) serves as a key modulator, governing stomatal closure, activating heat-responsive transcription factors, and regulating pathways related to antioxidants and osmoprotectants [49,50]. However, the responses to heat stress are orchestrated by a complex hormonal network that includes multiple signaling molecules.

Salicylic acid (SA) enhances thermotolerance by modulating ROS production and upregulating genes linked to pathogenesis-related proteins and heat shock proteins (HSPs) [51,52]. Importantly, SA plays a significant role in cross-tolerance mechanisms where abiotic and biotic stresses intersect [53,54,55].

Jasmonic acid (JA) contributes to thermal protection by promoting antioxidant activity and stabilizing proteins [56,57], thereby enhancing their resistance to heat stress. It has been demonstrated that exogenous application of methyl jasmonate (MeJA) enhances heat tolerance by modulating osmotic adjustment, strengthening the antioxidant defense system, and inducing the expression of JA-responsive genes [58].

It was also demonstrated that high-temperature stress during early pod set in soybeans increases ethylene (ET) production, which in turn triggers premature leaf senescence, induces oxidative stress, and significantly reduces photosynthesis, seed set, and yield. Foliar application of the ethylene perception inhibitor 1-methylcyclopropene (1-MCP) was shown to alleviate these adverse effects by reducing ethylene production and enhancing antioxidant defense mechanisms. Overall, this study demonstrated that ET plays a central role in HT-induced senescence and that 1-MCP application can improve physiological performance and yield under heat stress conditions [59].

It has been demonstrated that the exogenous application of the cytokinin derivative meta-topolin-9-(tetrahydropyran-2-yl)purine (mT9THP) enhances plant thermotolerance by modulating hormonal balance, activating stress-responsive genes, and stimulating the emission of protective volatile organic compounds (VOCs). The combination of mT9THP with heat acclimation (AHS: acclimation followed by heat stress) had the strongest protective effect, promoting antioxidant defense and VOC production, including lipoxygenase (LOX)-derived volatiles. These findings highlight the key role of VOCs in cytokinin- and acclimation-induced heat stress tolerance [60].

High-temperature (HT) stress can significantly reduce endogenous indole-3-acetic acid (IAA) levels, thereby impairing auxin signaling and resulting in decreased pollen viability. Exogenous application of IAA alleviates these negative effects by improving pollen viability and reducing oxidative damage under HT stress. These results highlight the crucial role of auxin in maintaining reproductive success and yield stability in plants exposed to high temperature stress [61].

Foliar application of brassinosteroid (β-sitosterol) alleviated the adverse effects of high temperature stress on snap bean (*Phaseolus vulgaris*) during late summer cultivation. Treatment with β-sitosterol improved vegetative growth, total yield, and pod quality, while also enhancing the accumulation of free amino acids in leaves and phenolic compounds in pods. These findings suggest that β-sitosterol can effectively contribute to improving heat stress tolerance and sustaining snap bean productivity under elevated temperature conditions [62].

As is commonly known, legume seeds are vital sources of protein, minerals, and bioactive compounds, including isoflavones, saponins, and phenolics, which provide significant nutraceutical and functional food benefits. However, under elevated levels of CO_2_ resulting from climate change and heat, the protein content in legumes such as soybeans, chickpeas, and faba beans may decline. Furthermore, the concentrations of iron and zinc could decrease, diminishing their nutritional value in human diets [63,64]. Conversely, stress conditions, including increased CO_2_ or heat, can increase the total phenolics and flavonoids in legumes. While this enhances their antioxidant capacity, it may also heighten antinutritional factors that hinder protein digestibility. These findings illustrate that climate warming has the potential to both reduce the nutritional quality of legumes and alter their bioactive composition. This highlights the need for breeding, agronomic, and food technology strategies that focus on preserving both protein content and beneficial compounds in legumes [65].

### 4.2. Higher Temperatures Can Increase Legume Vulnerability to Insect Pests and Exacerbate Threats from Toxin-Producing Fungi, Posing a Risk to Public Health

**Figure 2:**
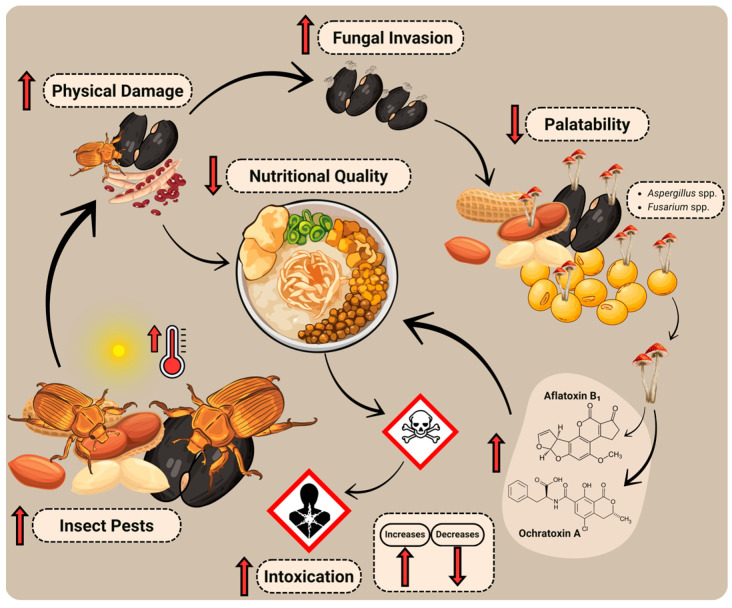
Rising temperatures increase the susceptibility of legumes to insect infestations and mycotoxin contamination, posing a threat to food safety and human health.

The interaction between elevated temperatures and biotic challenges is increasingly recognized as a crucial factor influencing the resilience of legumes and crop quality in the face of climate change (Figure 2). In leguminous plants, heat stress not only undermines their natural defense systems and sexual reproduction, for example, as shown in pea, common bean, lentil, and chickpea [66,67,68,69,70,71], but also creates conditions that promote pest development, reproduction, and feeding, as insects are poikilothermic organisms; their body temperature and metabolic activity are largely determined by the ambient environmental conditions [72].

Furthermore, heat stress directly influences the performance of insect pests, accelerating their development and enhancing their fecundity, which can lead to rapid population growth. This is particularly concerning for thermophilic pest species, such as the cowpea aphid (*Aphis craccivora*), the soybean looper (*Chrysodeixis includens*), and the western flower thrips (*Frankliniella occidentalis*), which exhibit increased feeding rates, fitness, and potential damage under warmer conditions [73]. Additionally, elevated temperatures can alter the types of volatile organic compounds (VOCs) released by plants, which may signal changes in insect behavior and attract their natural predators [74]. Such changes can disrupt tritrophic interactions among arthropod natural enemies, herbivores, and plants, thereby weakening the effectiveness of biological control mechanisms [75].

Recent studies suggest that simultaneous exposure to heat and herbivory triggers unique defense responses that are different from those elicited by each stressor alone. In plants experiencing both abiotic and biotic stress, a reconfiguration of primary metabolism occurs, including the transport of sugars and amino acids. The study by Zhong et al. [76] investigated the impact of transient high-temperature exposure on defense responses to herbivory in plants interacting with *Phthorimaea operculella* larvae. Larvae gained more weight on leaves subjected to both heat and herbivory than on leaves exposed to herbivory alone, indicating compromised plant defense under combined stress. Transcriptomic analyses revealed that high temperature altered the temporal pattern of gene expression from an evolutionary “hourglass” to a “vase” pattern, with early and late defense-related genes being suppressed. High temperature also impaired the accumulation of jasmonates and related defense metabolites following insect attack. Experiments with genetically modified plants confirmed that elevated temperatures weaken JA-mediated signaling and herbivore-induced defense. These insights highlight that the increase in pest outbreaks due to global warming is not just a matter of insect biology and agronomy; it also reflects the heightened vulnerability of legume crops resulting from higher temperatures, which may consequently reduce the nutritional value of the protein crop and threaten global food safety.

Stored-product insect pests, which develop more rapidly at higher temperatures, pose a significant threat to the nutritional quality of legume seeds intended for human consumption (Figure 2). Infestations by bruchid beetles, such as *Callosobruchus maculatus*, *Callosobruchus chinensis*, *Acanthoscelides obtectus*, and *Bruchus pisorum*, can inflict physical damage to seed tissues. This damage results in reduced concentrations of proteins, carbohydrates, and lipids, an increase in free fatty acid content (leading to rancidity), and a decline in both palatability and digestibility [77,78].

Furthermore, perforated or compromised seeds by insects become more susceptible to fungal invasion by *Aspergillus* and *Fusarium*, thereby increasing the risk of mycotoxin contamination, including aflatoxins (AFs) and ochratoxin A (OTA). Aflatoxin B_1_ (Figure 2), the most potent natural hepatocarcinogen, has been detected in groundnut products in Africa, sometimes at concentrations exceeding 100 µg/kg [79] (Akullo et al. 2025). The study by Jeong et al. [80] assessed AFs and OTA in *meju*, a fermented soybean food used as a raw material for the preparation of traditional fermented foods, including soy sauce and soybean paste, from three climatic regions of South Korea. AFs occurred in 10% of *meju* and 24.4% of soybean paste samples, while OTA was found in 50% and 48.9% of samples, with concentrations reaching 193.2 μg/kg and 26.3 μg/kg, respectively. Contamination was more frequent in the central region, likely due to reduced fungal competition during household fermentation. Although partial degradation occurred during processing, residual AFs and OTA persisted, highlighting the need for continuous monitoring of traditional soybean products [80].

## 5. Strategies for Pest Management in Leguminous Crops Under Climate Change, and Their Impact on Human Health

Effective pest management in leguminous cropping systems must evolve to address the dual challenges posed by global warming and the increasing frequency of pest outbreaks. Rising temperatures not only influence pest phenology, distribution, and population dynamics but also diminish the effectiveness of traditional control methods. In this context, sustainable pest management increasingly relies on integrated pest management (IPM) strategies that combine genetic, ecological, and technological innovations, all in alignment with the principles of climate-resilient agriculture [81,82,83].

Genetic resistance is fundamental to effective pest management in legumes. Current breeding programs are focused on developing cultivars that demonstrate resistance to both heat stress and significant insect pests, such as *A. craccivora*, *Maruca vitrata*, and *Helicoverpa armigera*. Advances in marker-assisted selection and genomic prediction are accelerating the integration of resistance genes and the identification of quantitative trait loci (QTLs) linked to thermotolerance and pest resistance [84,85,86,87]. Cultural practices can be strategically adapted to mitigate pest pressure under warming scenarios. Adjusting sowing dates to avoid synchrony with peak pest activity during heatwaves, increasing crop diversity, and employing trap cropping or push-pull strategies can enhance system resilience. Intercropping legumes with cereals or aromatic plants has been shown to disrupt pest colonization patterns, lower herbivore preference, and support beneficial insect communities [88,89,90,91,92,93,94].

Biological control provides a climate-smart alternative to traditional chemical pesticides, thereby enhancing food safety by reducing the health risks associated with pesticide residues. Synthetic pesticides often leave harmful residues on legumes, posing a threat to human health through ingestion and potential long-term exposure. In contrast, biological control agents—such as natural predators, parasitoids, or microbial agents—target specific pests without leaving harmful residues. This method significantly lowers the risk of human exposure to toxic substances [95].

The adoption of biological control methods significantly contributes to environmental sustainability by reducing chemical runoff and preserving beneficial organisms. This approach aligns with IPM strategies that prioritize ecological balance and reduce reliance on chemical inputs. Moreover, the implementation of biological control can enhance crop quality and yield stability by minimizing the risks associated with pesticide-induced phytotoxicity and the development of resistance. Not only does this support food security, but it also promotes the production of safer, more nutritious legume products [95,96].

Parasitoids, such as *Trichogramma* spp. and *Aphidius colemani*, and predators, like lady beetles and lacewings, are vital components of biological control. The effectiveness of these biocontrol agents can be influenced by temperature, underscoring the importance of selecting thermotolerant strains and implementing habitat management strategies. These strategies may include providing floral resources or creating microclimate buffers to support their activity and survival [97,98].

As stated previously, the increasing demand for sustainable pest management in leguminous crops has fostered the development and implementation of biological control agents, among which *Trichogramma* spp. have emerged as one of the most effective tools against lepidopteran pests. These minute egg parasitoid wasps, belonging to the family Trichogrammatidae, are extensively used worldwide due to their ability to parasitize the eggs of a wide range of economically significant moths and butterflies [99,100]. In legume agroecosystems, such as those of soybean, chickpea, cowpea, common bean, and faba bean, *Trichogramma* species have been particularly successful in managing key lepidopteran pests. These include *Anticarsia gemmatalis*, *Pseudoplusia includens*, *Spodoptera frugiperda*, and *H. armigera* [99,101,102].

Of particular interest is the application of *A. colemani* as a biological control agent against aphids in leguminous crops. *A*. *colemani* is a widely used parasitoid wasp that plays a crucial role in the biological control of aphid pests in various agricultural systems, including leguminous crops. This endoparasitoid is particularly effective against common aphid species such as *Aphis fabae*, *A. craccivora*, and *Myzus persicae*, which pose significant threats to faba bean, cowpea, and other Fabaceae crops [103,104]. In protected and open-field environments, *A. colemani* has demonstrated high parasitism rates and can effectively reduce aphid populations when released preventively or curatively [105,106]. Its rapid reproductive cycle, host specificity, and compatibility with IPM strategies make it an ideal agent for sustainable control, including aphid biological control in greenhouses [104]. Moreover, *A. colemani* is known for its adaptability to varying environmental conditions and compatibility with other natural enemies, including predatory insects such as *Coccinella septempunctata* and lacewings (*Chrysoperla* spp.) [107,108,109,110,111].

In conclusion, integrating biological control into pest management practices offers a safer and more sustainable approach to protecting protein crops, thereby enhancing food safety and promoting public health.

## 6. Climate Change: Dynamics of Legume–Pest Interactions: Regional Perspectives

Climate change is fundamentally transforming agricultural ecosystems globally, with leguminous crops being particularly affected. As critical contributors to food security through their roles in protein supply, nitrogen fixation, and soil fertility, legumes are increasingly threatened by the rapid emergence and spread of insect and mite pests. These alterations in pest dynamics are driven by rising temperatures, changes in precipitation patterns, and fluctuations in humidity levels.

### 6.1. South America

South America plays a central role in global food production [112,113,114], contributing significantly to Sustainable Development Goal 2—Zero Hunger [115]. However, as in other regions of the globe, this production is under threat when considering the current climate change scenario [116,117,118,119]. Droughts, extreme heatwaves, and rainfall variability, which can lead to flooding, rank among the primary challenges for agriculture [120,121,122]. In addition to directly affecting crop growth, development, and yields [123,124], these changes may alter the distribution and behavior of several agricultural pests [125,126,127,128]. Insects’ growth, development, reproduction, and survival, as well as their interactions with host plants, are strongly influenced by environmental modifications, particularly higher temperatures [81,125] and instability in rainfall frequency and distribution [126,127].

**Figure 3:**
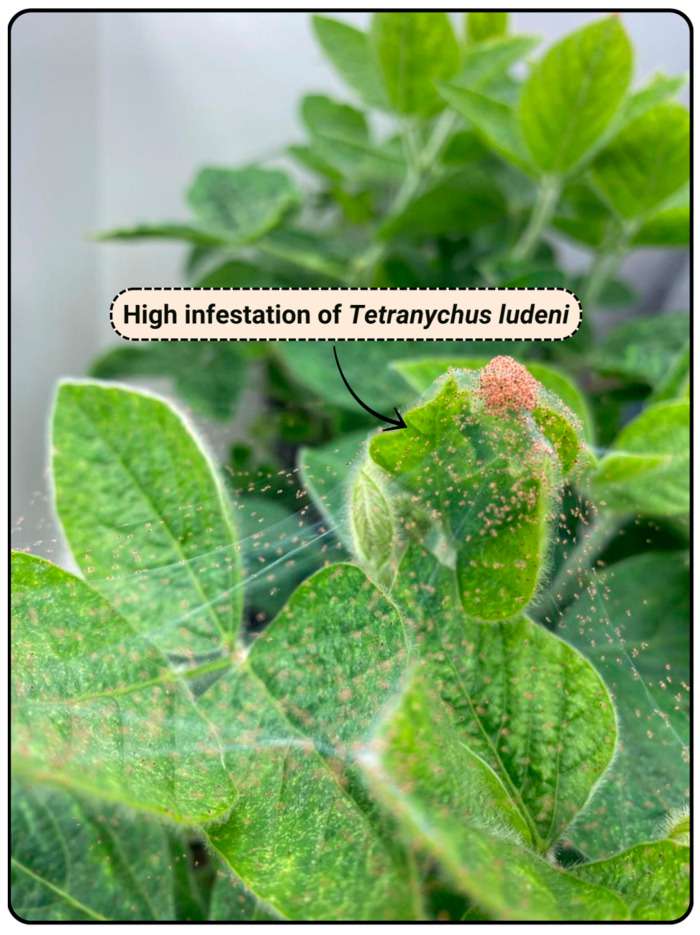
Laboratory stock colony of *Tetranychus ludeni* established from soybean plants collected in fields in Rio Grande do Sul, Brazil.

As already emphasized above, most insects and arthropods are poikilothermic, meaning they maintain body temperatures close to ambient conditions. Their development follows sigmoid curves, and both survival and fecundity are affected by thermal extremes. Although insect development and behavior are directly impacted by temperature, this factor also indirectly influences feeding activity. Thermal tolerance varies across species and ontogenetic stages and may be associated with the expression of HSPs [128]. Studies report pest population peaks coinciding with warmer and drier periods. In soybean fields in Rio Grande do Sul, Brazil, increased frequencies of phytophagous mites, such as *Tetranychus urticae* and *Tetranychus ludeni* (Figure 3), as well as *Mononychelus planki*, have been documented [129]. Conversely, *Polyphagotarsonemus latus* exhibits high mortality under elevated temperatures associated with low relative humidity.

Climate change can significantly alter the pest status of agricultural species, as temperature increases in historically cooler regions may expand the number of generations of species such as *S. frugiperda* [130]. High populations of *Euschistus heros* observed in soybean fields demonstrate the species’ adaptive capacity to warmer conditions. In these scenarios, both foliar and soil-dwelling herbivores may be affected. For example, temperature regime shifts appear to favor the soil weevil *Parapantomorus fluctuosus*, whereas drought favors *Elasmopalpus lignosellus*. Both species pose serious threats to legume agroecosystems, particularly damaging seedlings of soybeans, peanuts, and other crops. Similarly, *M. vitrata*, which attacks soybean pods, is favored by higher temperatures that accelerate development and stimulate oviposition. *Chrysodeixis includens* exhibits higher growth and developmental rates under warmer conditions, which explains the severe population outbreaks. Likewise, temperature increases up to 30 °C accelerate the life cycle of *Chloridea virescens*, expanding its relevance in soybean-producing regions. *H. armigera*, in turn, benefits from the combination of high temperatures and elevated humidity. Together, these pests have a significant impact on South American agriculture [131].

Importantly, while some species are favored, others may be negatively affected. For instance, *Anticarsia gemmatalis* does not withstand extreme temperatures [132]. Warming reduces its longevity and egg viability, while slowing development, without altering the sex ratio, indicating negative performance effects within the tested thermal range. These findings suggest that global warming may decrease its incidence in tropical regions but increase outbreaks in historically cooler areas, underscoring the need to consider evolutionary adaptation [132]. Other species also exhibit vulnerability: *Spodoptera cosmioides* displays reduced survival and egg viability at 32 °C [133], whereas *Spodoptera eridania* develops poorly at 34 °C. Among Coleoptera, *Cerotoma arcuata* and *Diabrotica speciosa* exhibited higher mortality in their juvenile stages at temperatures above 30 °C. In contrast, *Agrotis ipsilon* was able to tolerate temperatures ranging from 18 to 30 °C. *Nezara viridula* also shows reduced biological performance at higher temperatures, preferring cooler regions [134].

Beyond herbivores, climate change compromises biological control agents. Several natural predators and pathogens show reduced performance under extreme conditions, weakening integrated pest management strategies [135,136,137,138]. The predatory mite *Neoseiulus californicus*, for example, does not tolerate high temperatures coupled with low humidity [129]. Under such scenarios, chemical control is often intensified; however, pesticides may act as xenobiotics, exacerbating plant stress in response to herbivory, water imbalance, and other environmental factors, ultimately affecting human health [139]. Alternatively, bioactive peptides have been proposed as an innovative management tool [140]. Research in South America has explored peptides derived from protease inhibitors naturally present in legumes, showing promising results against lepidopteran pests in soybean [141,142]. These peptides may act synergistically with traditional biological agents, creating multiple barriers against emerging climate-driven pests.

Simulating the effects of climate change in experimental settings is crucial for designing efficient management alternatives. Future research should consider not only the direct impact on insect life cycles but also the potential for pest injury and plant responses under combined stress (pest attack plus elevated temperature). Some studies demonstrate that plants under drought or heat stress may be more susceptible to particular pests [143]. However, this pattern does not hold across all species [144,145]. Stress-induced plant defense mechanisms [146,147] may repel certain herbivores but simultaneously increase attractiveness to others, due to higher concentrations of soluble sugars, proteins, and amino acids [148,149,150]. These scenarios underscore the importance of considering regional variability in tropical countries like Brazil. Beyond temperature and humidity, other factors, such as atmospheric pressure, often overlooked, have been shown to alter insect circadian rhythms, significantly affecting foraging and reproduction in species like *Diabrotica speciosa* and *Macrosiphum euphorbiae* [151]. Elevated atmospheric CO_2_ and increased UV-B radiation also influence plant–insect interactions. Elevated CO_2_ may enhance herbivory by altering leaf C:N ratios, reducing defense compounds such as allantoin and protease inhibitors [152,153], and promoting greater insect fecundity and longevity. In contrast, UV-B radiation tends to have opposite effects, inducing the accumulation of phenolic compounds that decrease the feeding preference of pests such as *A. gemmatalis* [154].

Finally, available information concerning South America remains limited. As discussed, multiple studies indicate profound changes in pest and pathogen dynamics in agriculture. Thus, monitoring and preliminary studies to identify new herbivore–plant interactions are crucial for providing robust data to support informed decision-making and laying the groundwork for advanced management strategies under extreme climate conditions. The impact of climate change on agricultural pests often does not manifest immediately but rather across subsequent generations. Shifts in temperature or other environmental factors may affect development, fecundity, and survival rates, with long-term implications for population success. Furthermore, pest redistribution represents an additional challenge: areas currently unsuitable may, under global warming, become favorable for colonization and establishment. Consequently, more than short-term fluctuations in population dynamics, climate change is expected to alter pest occurrence patterns, with direct implications for integrated pest management, food security, and human nutrition at both regional and global scales.

### 6.2. India

In India, the incidence of the pod borer, a major pest of chickpea and pigeonpea (*Cajanus cajan*), has intensified due to warmer temperatures, which extend its breeding season and geographic range [155]. Similarly, the pulse beetle (*Callosobruchus* spp.), which damages stored pulses such as cowpeas, is becoming more problematic due to climate-related increases in temperature and humidity during storage [156]. Elevated temperatures and humidity create conditions conducive to rapid pest proliferation, resulting in significant quantitative losses and degradation of crop quality. Increased moisture levels in storage boost enzymatic activity in seeds and promote microbial growth, which can accelerate the breakdown of proteins, lipids, and micronutrients, ultimately diminishing the nutritional value of legumes. Moreover, these conditions promote the growth of certain fungi that produce mycotoxins, which pose significant health risks to humans upon consumption. Consequently, climate-induced changes in storage environments may jeopardize both the dietary quality and safety of legume seeds. This underscores the urgent need for enhanced post-harvest management strategies to mitigate pest infestations and prevent mycotoxin contamination.

### 6.3. Lithuania

Another interesting study investigated the impact of climatic factors on the population dynamics of *A. fabae* in faba bean fields located in a temperate European region [157]. The results demonstrated a strong positive correlation between rising maximum temperatures in the preceding 7 to 14 days and increased aphid abundance, with correlation coefficients ranging from 0.738 to 0.826. In 2023, temperature data from the week before monitoring explained over 60% of the variation in aphid populations across two cultivars, ‘Vertigo’ and ‘Fuego’, highlighting temperature as the primary driver of aphid outbreaks. Although rainfall showed some short-term negative effects on aphid numbers, temperature remained the most consistent predictor of infestation severity. The authors conclude that climate warming, especially the increase in maximum daily temperatures, is contributing to more frequent and severe *A. fabae* outbreaks, underscoring the need for temperature-based forecasting and management strategies in faba bean cultivation [157].

### 6.4. Greece

Field trials conducted by Tsialtas and Irakli [158] demonstrated that temperature has a significant influence on the activity and reproduction of *B*. *rufimanus*. The authors observed that adult weevils become active when temperatures exceed 15 °C, a factor that can directly affect infestation levels.

### 6.5. France, Belgium, and Luxembourg

Findings reported by Carrillo-Perdomo et al. [159] in France emphasized the role of temperature and humidity in shaping insect oviposition dynamics and egg viability. In Belgium, Segers et al. [160] highlighted how weather-driven variations affect pest pressure and seed damage, also emphasizing temperature as a crucial element in the damage caused by pests. Vitali et al. [161] examined the long-term effects of climate change and land-use alterations on the phenology and distribution of longhorn beetles (Cerambycidae) in Luxembourg over the past 150 years. Researchers analyzed a dataset of 71 species recorded between 1864 and 2014. Results indicate that rising annual temperatures have led to earlier beetle appearances, with species observed on average 8.2 days earlier in the year post-1980. Notably, spring warming (March–June) advanced beetle emergence by approximately 9.6 days per 1 °C increase in temperature. Additionally, the study found a net gain in species richness over the last four decades, with 11 species recorded exclusively after 1997, characterized by earlier annual appearances. Smaller-bodied beetles tend to appear earlier than larger ones. Shifts in beetle phenology were not correlated with species Red List status, suggesting that both climate change and land-use changes have influenced these patterns. The study highlights that the combined effects of climate and land-use changes have resulted in significant shifts in the occurrences of longhorn beetles in Luxembourg, particularly affecting species with continental climate niches.

### 6.6. Germany

The study by Hannigan et al. [162] investigated how temperature, along with light conditions and beetle weight, affects the movement and feeding behavior of the large lupine beetle (*Sitona gressorius*). Using video tracking of 384 beetles at six different temperature levels (10–35 °C) under both light and dark conditions, the researchers assessed flight probability, movement speed, displacement, activity time, and feeding rate. They found that flight probability, speed, displacement, and diffusion coefficient increased significantly with higher temperatures, especially under low light conditions. Notably, beetle activity and diffusion peaked at both low and high temperatures, with a marked dip around 20 °C, suggesting a non-linear thermal response. The feeding rate also increased consistently with temperature, reaching its maximum at 35 °C. Hidden Markov modeling revealed that beetles spent more time in active movement states as the temperature increased, with the highest diffusion occurring near 35 °C. These findings highlight that temperature is a critical driver of beetle dispersal and feeding, and models that assume constant diffusion may underestimate pest spread under fluctuating thermal conditions.

### 6.7. Algeria

According to a study by Hamani and Medjdoub-Bensaad [163], temperature plays a significant role in the development and life cycle of the bean weevil, *B. rufimanus*. The egg-laying period varied depending on environmental conditions, lasting 28 days on the Seville variety and 35 days on the field bean variety, indicating that higher temperatures may accelerate oviposition and thus influence population growth. Additionally, the complete development cycle from egg to adult emergence took approximately 4 months and 16 days for the Seville variety and 4 months and 18 days for the Field bean, suggesting that temperature fluctuations impact the duration of the pest’s life cycle. These findings suggest that rising temperatures due to climate change may reduce the development time of *B. rufimanus*, potentially leading to increased population densities and more severe damage to broad bean crops.

### 6.8. Australia

The study on the potential for biological control of the vegetable leafminer, *Liriomyza sativae*, in Australia highlights the critical role of temperature in shaping both the pest and its natural enemies’ dynamics [164]. Since its detection on the Australian mainland in 2015, *L. sativae* has posed a significant threat to horticultural and nursery crops, including leguminous plants such as beans and peas, which are among its many host species. Temperature influences the population growth of *L. sativae* as well as the effectiveness of parasitoid wasps such as *Diglyphus isaea* and *Hemiptarsenus varicornis*, which are key biological control agents. However, extreme temperatures can reduce the survival and reproductive success of these parasitoids, potentially limiting their ability to control the pest. Therefore, integrated pest management strategies that consider the temperature effects on both *L. sativae* and its parasitoids are essential for effective control of this pest in Australian and global cropping systems [164].

## 7. Conclusions

Legumes, belonging to the Fabaceae family, are essential for promoting global food security and enhancing human health. Their dual function as nutrient-dense foods and sustainable crops that can fix biological nitrogen positions them as pivotal in strategies aimed at achieving nutritional adequacy and ecological resilience. Rich in proteins, complex carbohydrates, dietary fiber, essential fatty acids, and a diverse range of micronutrients, legumes significantly contribute to mitigating widespread nutritional deficiencies and alleviating the burden of diet-related chronic diseases. Additionally, their high levels of bioactive compounds, including polyphenols, carotenoids, and bioactive peptides, reinforce their status as functional foods with potential preventive effects against metabolic disorders, cardiovascular diseases, and inflammation. Nevertheless, the stability of this crucial nutritional resource is increasingly threatened by global warming. Projected increases in temperature, altered precipitation patterns, and elevated CO_2_ levels are anticipated to decrease the protein, iron, and zinc content of legume seeds, while also altering their bioactive composition. Such changes may have substantial implications for populations that depend on legumes as a primary source of protein. Furthermore, the rise in insect pest pressures and the proliferation of mycotoxin-producing fungi, driven by climate change, present additional risks that could jeopardize both yield stability and food safety. To ensure the safeguarding of high-nutritional-quality legumes for future generations, a comprehensive approach is imperative. Innovations in breeding and biotechnology should prioritize the simultaneous enhancement of heat tolerance, pest resistance, and nutritional stability. The adoption of integrated pest management strategies, particularly those that incorporate biological control measures, will provide pathways to reduce pesticide use and mitigate associated health risks. Moreover, it is essential to valorize by-products from legume processing, which are abundant in fiber and phytochemicals, as they can be integrated into functional food formulations, thus promoting a circular bioeconomy and reducing waste within the food system. In conclusion, ensuring the resilience of legumes in the context of climate change constitutes not only an agricultural imperative but also a vital aspect of nutritional and public health. By integrating plant science, nutrition research, and sustainable agricultural practices, stakeholders can effectively protect the nutritional integrity of legumes while fostering safer and healthier dietary options.

## Data Availability

No new data were created or analyzed in this study. Data sharing is not applicable to this article.
